# Significance of peak height velocity as a predictive factor for curve progression in patients with idiopathic scoliosis

**DOI:** 10.1186/1748-7161-10-S2-S5

**Published:** 2015-02-11

**Authors:** Masaaki Chazono, Takaaki Tanaka, Keishi Marumo, Katsuki Kono, Nobumasa Suzuki

**Affiliations:** 1 Department of Orthopaedic Surgery, Utsunomiya National Hospital, Tochigi, 329-1193, Japan; 2 Department of Orthopaedic Surgery, The Jikei University School of Medicine, Tokyo, 105-8461, Japan; 3 Department of Orthopaedic Surgery, Eiju General Hospital, Tokyo, 110-0015, Japan; 4 Scoliosis Center, Medical Scanning Tokyo, Tokyo, 103-0027, Japan

## Abstract

**Background:**

Much attention has been paid to peak height velocity (PHV) as a possible predictor of curve progression in patients with idiopathic scoliosis (IS). The aim of this study was to analyze the relationship between the magnitude of the Cobb angle at PHV and scoliosis progression, defined as having surgery prior to skeletal maturity in female patients with IS.

**Methods:**

A retrospective review identified 56 skeletally immature female IS patients who were followed until maturity. The mean age and the mean pubertal status at the initial visit were 10 years and 24 months before menarche respectively, with a follow-up period of 5 years. They were divided into two groups: non-surgery group (NS) and surgery group (S), depending on their treatment method in use at the final follow-up visit. Surgery group was defined as an ultimately having surgery due to Cobb angle greater than 45 degrees prior to skeletal maturity regardless of conservative management. Height measurements were recorded at each visit; height velocity was calculated as the height change, in cm, divided by the time interval, in years. The PHV, chronological age at PHV (APHV), height at PHV (HPHV), and final height (FH) were determined for each group. In patients with Cobb angle greater than 30 degrees, the corrected height was calculated by Kono formula and corrected height velocity values were provided. The sensitivity, specificity, and area under the curve (AUC) of the receiver-operating -characteristic (ROC) analysis were calculated to predict spinal curve progression for various Cobb-angle cutoff values at PHV.

**Results:**

The corrected PHV had a mean value of 8.5 and 8.9 cm/year in the NS-group and S-group, respectively. The APHV was 11.9 and 11 years, the corrected HPHV was 152.9, and 149.3 cm, and the corrected FH was 159.9 and 159.3 cm, respectively. When a Cobb angle of 31.5 degrees was at PHV, ROC analysis revealed 78% sensitivity, 82% specificity, and an AUC of 0.93, acceptable values for curve progression in patients with IS.

**Conclusions:**

These findings indicate that 31.5 degrees of spinal curvature when patients are at PHV is a significant predictive indicator for progression of the curve to a magnitude requiring surgery. We suggest that the curve-progression risk assessment in patients with IS should include PHV, along with measures of skeletal and non-skeletal maturities.

## Background

Recently, much attention has been paid to peak height velocity (PHV) as a possible predictor of curve progression in patients with idiopathic scoliosis (IS) [1-3]. The aim of this study was to analyze the relationship between the magnitude of the Cobb angle at PHV and scoliosis progression, defined as having surgery prior to skeletal maturity in female patients with IS.

## Methods

With institutional review board of Utsunomiya National Hospital approval, we retrospectively reviewed the data regarding patients with idiopathic scoliosis. Inclusion criteria for this study were: patients who visited our scoliosis clinics who were immature at the time of the initial visit, had a Risser score of 0, a digital skeletal age of hand of 2 or 3, and had not yet reached menarche or their height growth spurt. Fifty-six skeletal immature females were enrolled in this study and were followed until maturity. The mean age and the mean pubertal status at the initial visit were 10 years and 24 months before menarche respectively, with a follow-up period of 5 years. They were divided into two groups: non-surgery group (NS) and surgery group (S), depending on their treatment method in use at the final follow-up visit. Surgery group was defined as an ultimately having surgery due to Cobb angle greater than 45 degrees prior to skeletal maturity regardless of conservative management. Height measurements were recorded at each visit; height velocity was calculated as the height change, in cm, divided by the time interval, in years between visits of 6 and 12 months, averaging at 8 months. The PHV, chronological age at PHV (APHV), height at PHV (HPHV), and final height (FH) were determined for each group. In patients with Cobb angle greater than 30 degrees, the corrected height was calculated by Kono formula and corrected height velocity values provided. According to the method of Kono formula, the following correction equation for body height by Cobb angles: Y=0.6X+2.6 (mm) where X=∑(Cobb-30) = (Cobb*_1_*-30) + (Cobb*_2_*-30) +…+ (Cobb*_n_*-30) [4]. The sensitivity, specificity, and area under the curve (AUC) of the receiver-operating -characteristic (ROC) analysis were calculated to predict spinal curve progression for various Cobb-angle cutoff values at PHV.

## Results

### Corrected PHV (peak height velocity)

The corrected median PHV values were 8.5 and 8.9 cm/year in the NS-group and S-group, respectively. Height velocity greater than 7 cm/year suggests the onset of PHV (Table 1).

**Table 1:** The summary of corrected peak height velocity, chronological age at peak height velocity, corrected height at peak height velocity, and corrected final height between non-surgery and surgery groups.

	NS-group	S-group
Corrected PHV (cm/year)	8.5±2.0	8.9±2.1
APHV (years old)	11.9±1.0	11±1.1

Corrected HPHV (cm)	152.9±5.7	149.3±8.3

Corrected FH (cm)	159.9±5.7	159.3±7.2

Note: Values are presented as means and SDs.

Abbreviation: PHV: Peak height velocity, APHV: Chronological age at PHV, HPHV: Height at PHV, FH: Final height; NS-group = non-surgery group, S-group = surgery group

### APHV (chronological age at PHV)

The APHV was 11.9 and 11 years in the NS-group and S-group, respectively. APHV usually occurs between 11 and 12 years of age (Table 1).

### Corrected HPHV (height at PHV)

The corrected HPHV was 152.9 and 149.3 cm in the NS-group and S-group, respectively. In Japanese female patients, 150cm of height correlates with the timing of PHV (Table 1).

### Corrected FH (final height)

The corrected FH was 159.9 and 159.3 cm in the NS-group and S-group, respectively. The spinal longitudinal length by correction surgery was anticipated in the surgery group, however, the final height between the 2 groups was not significantly different. This finding may account for the cessation of spinal longitudinal growth within the fused levels in growing children.

### Receiver-operating-characteristic (ROC) analysis

**Figure 1:**
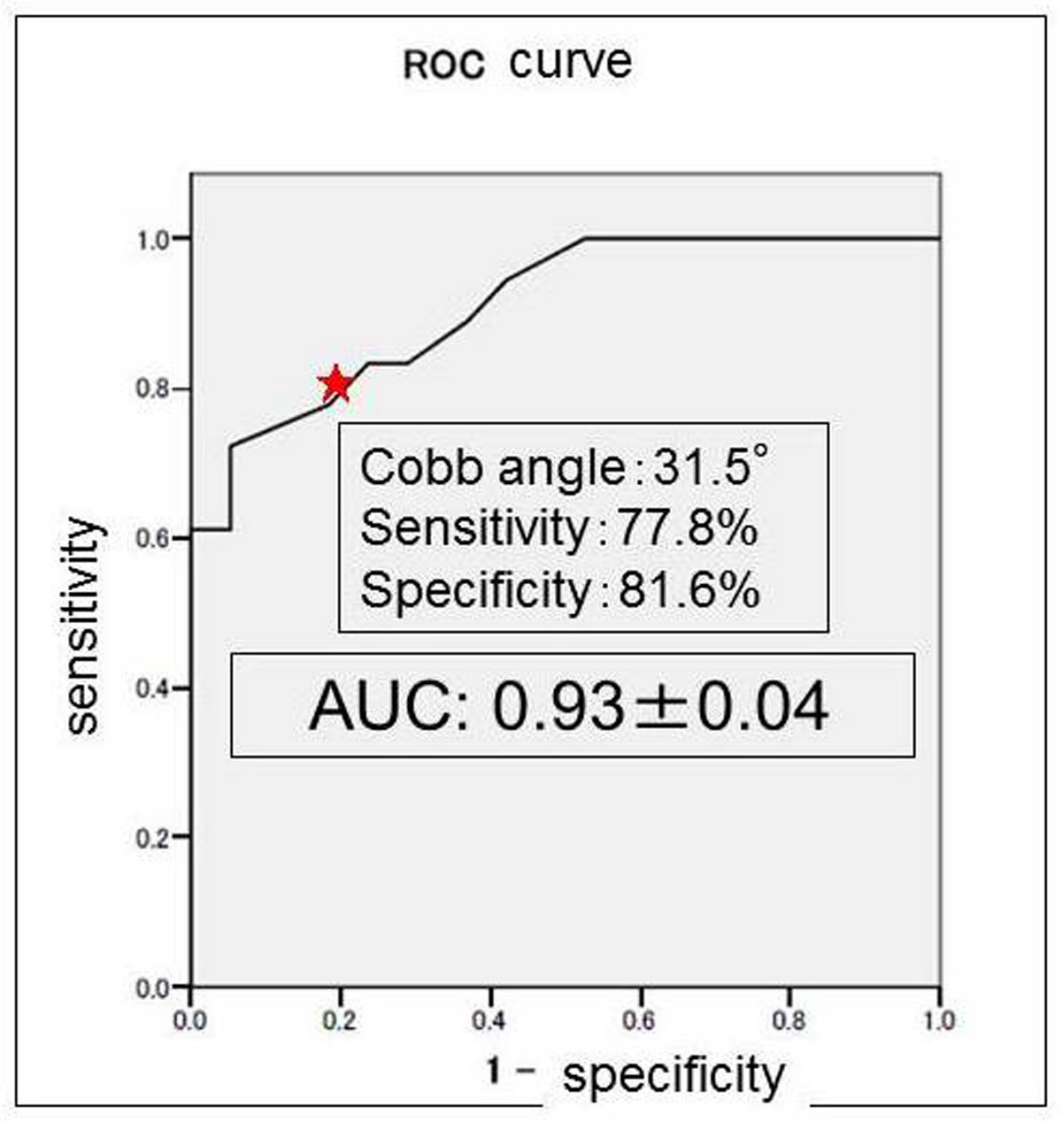
**Receiver-operating-characteristic curve for various Cobb-angle cutoff values at peak height velocity.** Star indicates that 78% sensitivity, 82% specificity, and AUC of 0.93 at the Cobb angle of 31.5 degrees.

**Figure 2:**
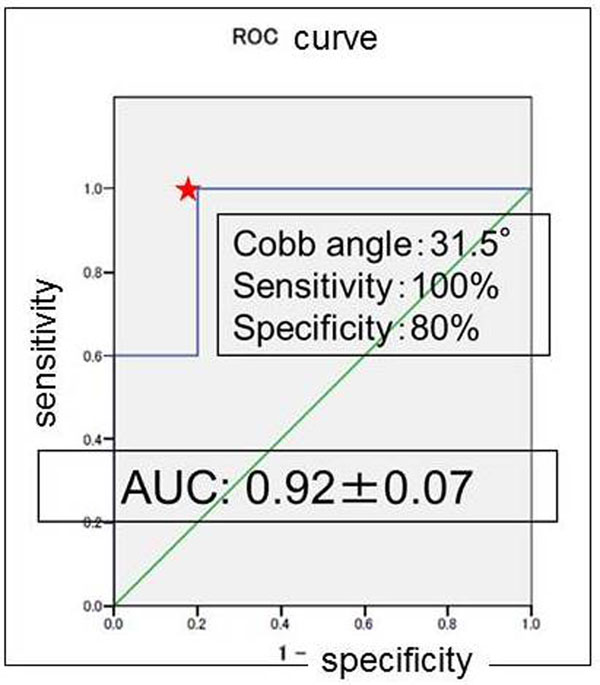
**Receiver-operating-characteristic curve for various Cobb-angle cutoff values at peak height velocity in single curve (n=15).** Star indicates that 100% sensitivity, 80% specificity, and AUC of 0.92 at the Cobb angle of 31.5 degrees.

**Figure 3:**
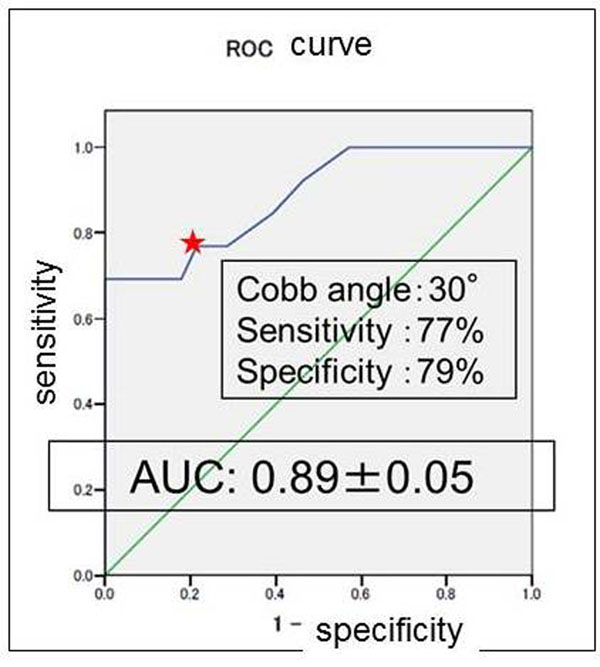
**Receiver-operating-characteristic curve for various Cobb-angle cutoff values at peak height velocity in double curve (n=41).** Star indicates that 77% sensitivity, 79% specificity, and AUC of 0.89 at the Cobb angle of 30 degrees.

When a Cobb angle of 31.5 degrees was used as the cutoff for determining which patients underwent surgery, ROC analysis revealed 78% sensitivity, 82% specificity, and an AUC of 0.93, acceptable values for curve progression in patients with IS (Fig. 1). In addition, 56 subjects were divided into two categories between patients who have single curve and double curves. In the single curve group (*n*=15), the optimal cutoff values was Cobb angle of 31.5 degrees with AUC of 0.92 (Fig. 2), whereas Cobb angles of 30 degrees with AUC of 0.89 in the double curves group (*n*=41) (Fig. 3).

## Discussion

**Figure 4:**
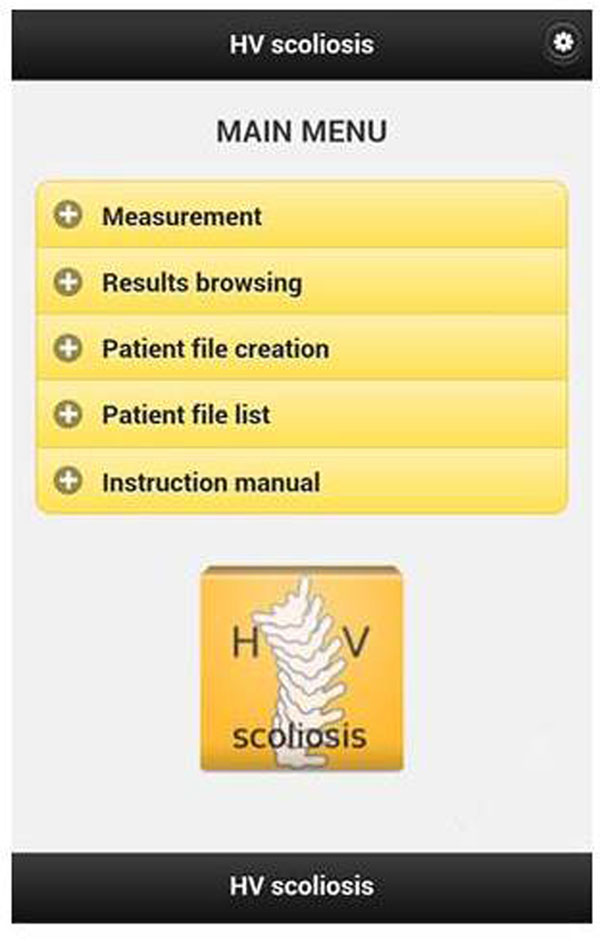
A mobile application software “HV scoliosis”.

Various skeletal and non-skeletal maturity indicators have been advocated as prognostic factors for curve progression [5-8]. For two decades, Lonstein and Carlson’s classification has been utilized to predict curve progression in adolescent idiopathic scoliosis [9]. However, its predictive accuracy is not always very good. Little et al. initially demonstrated that most curves progressed maximally at the time of PHV [1]. They found that 60 of 88 patients had a scoliosis curve of greater than 30 degrees at PHV, and in 50 of 60 patients, the curve had progressed to 45 degrees or greater. Ylikoski reported that progression was most notable in cases with a growth velocity of greater than 2 cm/year [10]. Our previous study using logistic regression analysis demonstrated that height velocity was the only significant independent variable for curve progression [11]. Therefore, not only PHV but also height velocity was considered to be a reliable marker for the prediction of remaining growth and progression of scoliosis. PHV is only identified retrospectively so that the value is not able to be known at the first visit of outpatient clinic. Our previous study also investigated a significant relationship between the height velocity and skeletal and non-skeletal maturity indicators [11]. When all the digital epiphyses began to curl over the edge of the metaphyses, the stage of capped epiphysis coincided with the growth peak greater than 7 cm/year. Therefore, hand X-ray is an alternative maturity indicator for evaluating the height growth peak. Sanders et al. also reported that if a patient has uncapped phalangeal epiphyses, then she probably has not reached PHV and if her phalangeal epiphyses are closed, she is likely to already have achieved PHV [3]. Recently, we have developed the mobile application software “HV scoliosis”, which is downloadable by Google play or App store to quickly calculate height velocity values in the clinical practice without a complex formula. Japanese version was released on April, 2013, following English version on January, 2014 (Fig. 4). Ease and prompt acquisition of PHV values in the clinical practice will be expected to be useful for determining whether scoliotic deformity progresses or not. From the present study, knowing the timing of the growth peak and the magnitude of the Cobb angle provides valuable information about the likelihood of the curve progression to a magnitude of requiring surgery.

## Conclusions

These findings indicate that 31.5 degrees of spinal curvature when patients are at PHV is a significant predictive indicator for progression of the curve to a magnitude requiring surgery. We suggest that the curve-progression risk assessment in patients with IS should include PHV, along with measures of skeletal and non-skeletal maturities. This is the extended abstract of IRSSD 2014 program book [12].

## List of abbreviations used

PHV: peak height velocity; IS: idiopathic scoliosis; HV: height velocity; APHV: chronological age at PHV; HPHV: height at PHV; FH: final height; ROC: receiver-operating-characteristic analysis; AUC: area under curve

## Competing interests

The authors declare that they have no competing interests, including no financial competing interests with Choi Dongsool, who developed a mobile application software “HV scoliosis” as a system engineer.

## Authors’ contributions

MC designed and coordinated the study, performed data analysis, and drafted the manuscript. TT, KK, and NS helped to draft the manuscript. All authors read and approved the final manuscript.

## Declarations

Publication charges for this collection were funded by IRSSD 2014 Sapporo.

This article has been published as part of *Scoliosis* Volume 10 Supplement 2, 2015: Research into Spinal Deformities 9: Short Papers from the IRSSD 2014 Meeting. The full contents of the supplement are available online at http://www.scoliosisjournal.com/supplements/10/S2.
